# Causal role of the inferolateral prefrontal cortex in balancing goal-directed and habitual control of behavior

**DOI:** 10.1038/s41598-018-27678-6

**Published:** 2018-06-20

**Authors:** Mario Bogdanov, Jan E. Timmermann, Jan Gläscher, Friedhelm C. Hummel, Lars Schwabe

**Affiliations:** 1 Department of Cognitive Psychology, Institute for Psychology, University of Hamburg, Hamburg, Germany; 2 Department of Neurology, University Medical Center Hamburg-Eppendorf, 20246 Hamburg, Germany; 3 Institute for Systems Neuroscience, University Medical Center Hamburg-Eppendorf, 20246 Hamburg, Germany; 4 Defitech Chair of Clinical Neuroengineering, Swiss Federal Institute of Technology (EPFL), 1202 Geneva, Switzerland

## Abstract

Successful adaptation to complex environments depends on the balance of at least two systems: a flexible but slow goal-directed system encoding action-outcome associations and an efficient but rigid habitual system linking responses to preceding stimuli. Recent evidence suggests that the inferolateral prefrontal cortex (ilPFC), a region well known to contribute to cognitive control processes, may play a crucial role in the balance of goal-directed and habitual responding. This evidence, however, comes mainly from correlational data and whether the ilPFC is indeed causally involved in the goal-directed vs. habitual control of behavior is unclear. Here, we used neuro-navigated theta-burst stimulation (TBS) to either inhibit or enhance right ilPFC functionality before participants completed an instrumental learning task designed to probe goal-directed vs. habitual behavioral control. TBS did not affect overall learning performance. However, participants that had received inhibitory TBS were less able to adapt their behavior to altered task demands, indicating a shift from goal-directed towards more habitual control of behavior. Sham or excitatory TMS groups showed no such effect and were comparable in their performance to an unstimulated control group. Our findings indicate a causal role of the ilPFC in the balance of goal-directed vs. habitual control of behavior.

## Introduction

Successful adaptation to varying environments requires an intricate balance of thoughtful deliberation and efficient responding. To achieve this balance, behavior can be controlled by distinct systems, a goal-directed or model-based system encoding the relationship between actions and their consequences and a habitual or model-free system involved in the formation of stimulus-response associations^1–3^. Converging lines of evidence from lesion studies in rodents and human neuroimaging studies implicated mainly the orbitofrontal cortex and dorsomedial striatum in goal-directed action^4–8^, whereas the dorsolateral striatum was identified as a key locus of habitual responding^9–12^.

Very recent evidence points to another region that may be critical for the goal-directed vs. habitual control of behavior, the inferolateral prefrontal cortex (ilPFC)^13^. Classically, the ilPFC has been associated with cognitive control processes, including the maintenance and release of inhibitory control^14–17^. While these functions are closely linked to goal-directed behavior, the ilPFC has recently been argued to act as an arbitrator that determines to what extent goal-directed and habit systems can govern behavior^13^. Specifically, it is argued that the arbitration mechanism works by reducing both the activity of brain areas involved in habitual control as well as their connectivity to each other and the arbitrator region in cases in which the arbitrator deems that behavior should be guided by the goal-directed system. However, the claim that the ilPFC is critically involved in the balance of goal-directed and habit behavior is based on correlational neuroimaging data and whether the ilPFC plays a causal role in the goal-directed vs. habitual control of behavior is currently unknown.

**Figure 1:**
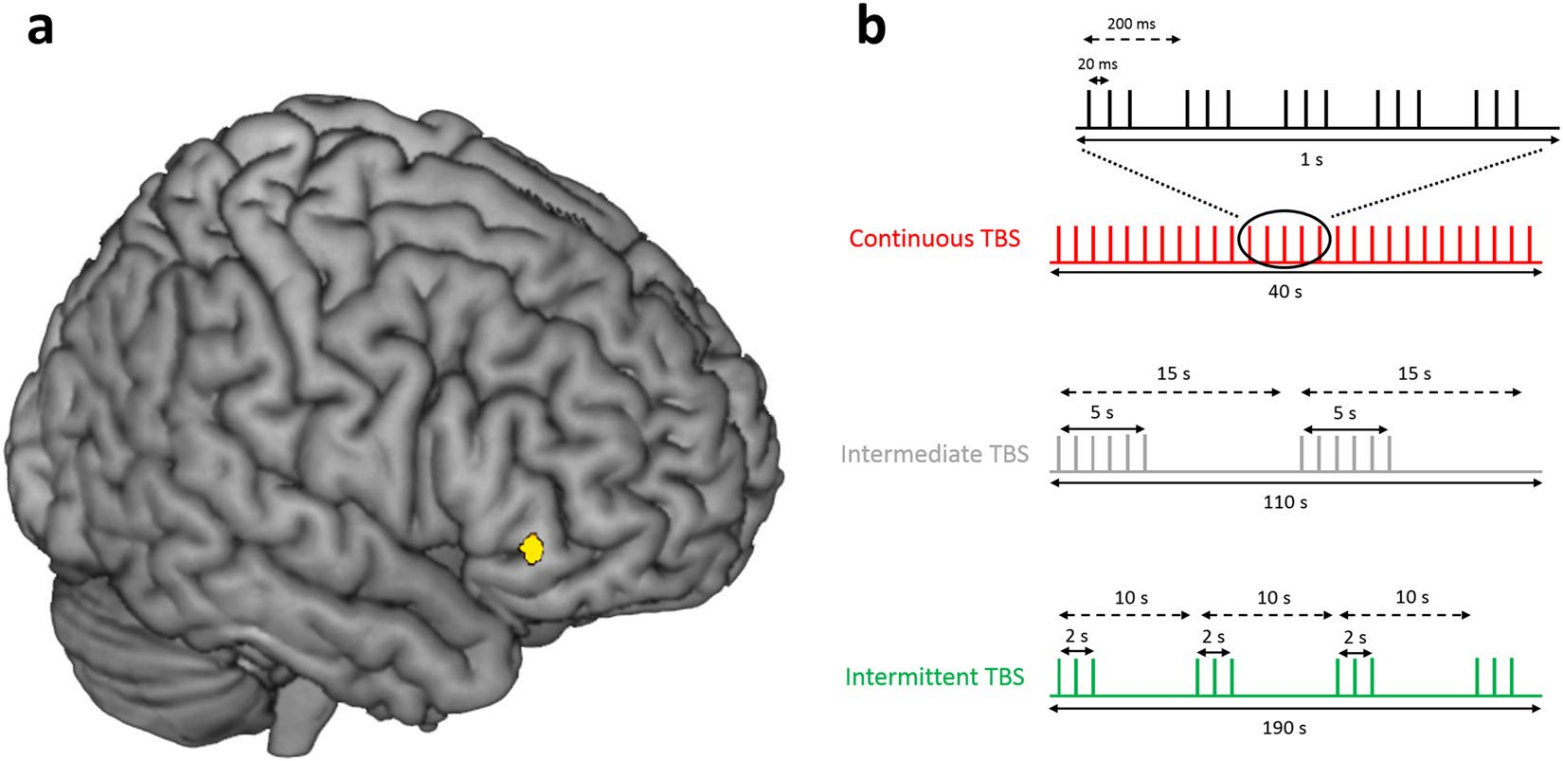
Theta-burst stimulation protocol. (**a**) Using an individual anatomical MRI image and a stereotaxic frameless Brainsight neuronavigation system, theta burst stimulation (TBS) was applied over the right inferior lateral prefrontal cortex (MNI: x = 48, y = 35, z = −2). Coordinates were chosen based on former neuroimaging findings, suggesting that the ilPFC is involved in the interplay of habitual and goal-directed behavior^13^. (**b**) TBS pulses were delivered in triple bursts, consisting of three magnetic pulses at an interval of 50 Hz. Bursts were delivered at an interval of 5 Hz. Participants received in total 600 pulses, in one of three TBS protocols that served as either inhibitory (cTBS), excitatory (iTBS) or sham stimulation (imTBS)^18^. Continuous (**c**) TBS consisted of 40 seconds continuous stimulation. Intermediate (im)TBS consisted of 5 second trains of active stimulation followed by a 10 second pause, lasting for a total of 110 seconds. Intermittent (i)TBS consisted of 2 second trains of active stimulation followed by an 8 second pause, lasting for a total of 190 seconds. In the no-stimulation control condition, participants did not receive any stimulation.

**Figure 2:**
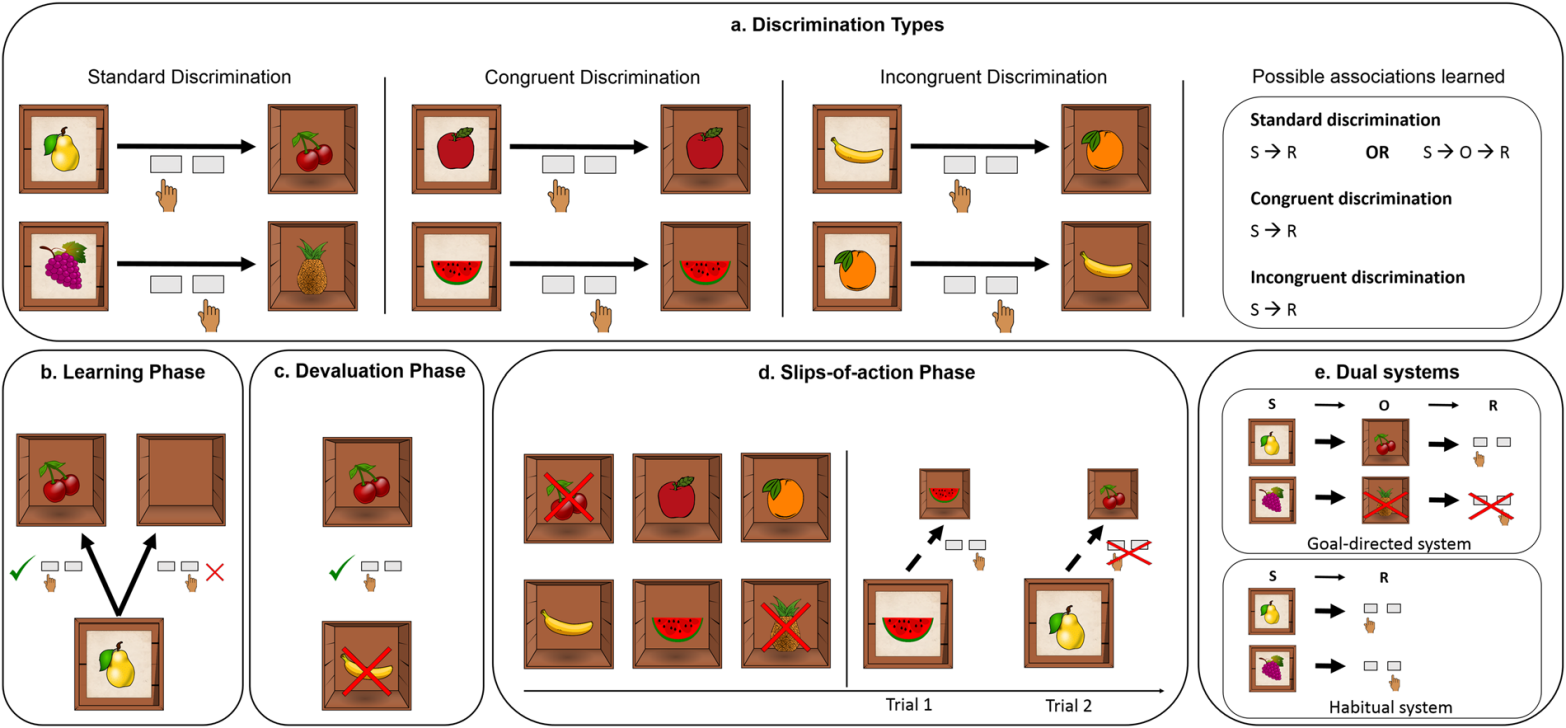
Overview of the Instrumental Learning Task (ILT). Participants were presented with 6 stimulus pairs consisting of fruit pictures. These pictures could serve as discriminative cue stimuli (depicted on top of a closed box) or outcome stimuli (depicted inside an open box). Stimuli pairs were linked by responses, which, if correct, would lead from cue to outcome. (**a**) Stimuli pairs belonged to one of three discrimination conditions: standard, congruent, and incongruent. In the congruent condition, cue and outcome were identical. In the incongruent discrimination condition, different fruits served as cue and outcome. However, fruits reversed their role as cue and outcome across trials, demanding opposite responses depending on which fruit served as a cue. In the standard discrimination condition, discriminative cue stimuli and outcome stimuli were unique fruits. (**b**) In the 9-block learning phase, participants were asked to learn the associations between the fruit stimulus pairs and the correct responses by trial-and-error. A correct response led to the outcome fruit and rewarded points. An incorrect response resulted in an empty box and no points. (**c**) In the outcome devaluation phase, participants were presented with two formerly rewarded outcome fruits associated with opposite responses. However, one fruits was now declared devalued, indicated by a red cross. Participants were asked to press the button that was associated with the still valuable reward. (**d**) In the 6-block slips-of-action phase, participants were first presented with an overview of all formerly rewarded outcome stimuli at the beginning of each block, two of which were now devalued. Subsequently, they were again presented with the discriminative cue stimuli. Participants were instructed to only show the correct response to cues with still valuable outcomes. If the outcome had been devalued, participants should refrain from responding at all. (**e**) If participants used the goal-directed system (i.e. S-O-R associations), responses should have been adapted to the actual value of the outcome stimuli. If, however, participants responded habitually (i.e. using S-R associations) they should show responses to stimuli with devalued outcomes (so-called slips-of-action). In this phase, correct responses would earn points whereas slips-of-action would lead to a subtraction of points. Pictures of fruits were taken from free online sources (pixabay.com, openclipart.org and clker.com).

To test whether the ilPFC is indeed causally involved in the goal-directed vs. habitual control of behavior, we employed neuro-navigated, sham-controlled Theta Burst Stimulation (TBS), a non-invasive technique that uses magnetic fields to directly decrease (continuous TBS) or increase (intermittent TBS) brain activity in the targeted region^18^ (Fig. 1). TBS was applied over the right ilPFC, exactly over the location postulated to modulate the balance of goal-directed and habitual action^13^, before participants completed an instrumental learning task (ILT), designed to test the degree of habitual vs. goal-directed control of behavior^19,20^. Throughout the ILT, participants were presented with fruit stimulus pairs, serving either as discriminative stimuli or as outcomes. The stimulus pairs were linked by responses that, if correct, led from a discriminative stimulus to an outcome stimulus. Goal-directed action is indicated by the formation of stimulus-outcome-response (S-O-R) associations, whereas habitual behavior is reflected in simpler stimulus-response (S-R) associations. Stimulus pairs belonged to one of three different discrimination conditions (Fig. 2a): congruent, incongruent, and standard. In the congruent condition, the same fruit served as discriminative stimulus and outcome. Thus, participants could rely on simple O-R associations in these trials^20^. In the incongruent condition, stimulus pairs reversed their status as discriminative stimulus or outcomes across different trials. To avoid response conflict, participants in the incongruent condition usually rely on habitual S-R associations^19^. Finally, in the standard condition, correct responses towards discriminative stimuli lead to unique outcome stimuli. The relative goal-directed vs. habitual control of behavior was tested in a subsequent ‘slips-of-action test’ requiring participants to abstain from responding to devalued actions. We hypothesized that, compared to sham or intermittent TBS, continuous TBS interfering with ilPFC functioning would result in less goal-directed behavior and hence more slips of action in the critical third stage of the experiment, either due to a direct impairment of the goal-directed system or indirectly via an impaired arbitrator mechanism, hindering the downregulation of the habit system. This impairment should be particularly pronounced in standard trials that can be controlled both by the goal-directed and the habitual system^20,21^. Although data on behavioral facilitation after magnetic brain stimulation in healthy subjects are not very consistent^22–27^, we predicted that increased brain activity through intermittent TBS might even facilitate the goal-directed control of action. Even though intermediate TBS is considered to be a sham condition^18,28^, we also included a no-stimulation control group to control for general effects of TBS. Importantly, we did not expect any differences between the groups in neither the learning phase nor the devaluation phase of the ILT.

## Results

### Learning phase

In the initial *learning phase* of the ILT, participants were asked to learn associations between fruit pairs and responses (Fig. 2b). Participants learned these associations very well, as indicated by a significant increase in correct responses across blocks (*F*_(4.621,281.303)_ = 78.657, *p* < 0.001, η²_p_ = 0.56). Although there was a marked increase in performance across trials in all discrimination conditions (all *F* > 24.648, all *p* < 0.001, all η²_p_ > 0.29), performance differed, as expected, for the three discrimination conditions (*F*_(2,122)_ = 26.927, *p* < 0.001, η²_p_ = 0.31): stimulus pairs in the congruent condition were learned best (vs. standard: *p* = 0.044, vs. incongruent: *p* < 0.001) and stimulus pairs in the incongruent condition were learned worst (vs. standard: *p* < 0.001). Learning performance was generally comparable in the four experimental groups (main effect: F_(3,61)_ = 0.571, *p* = 0.636, η²_p_ = 0.03), both across discrimination conditions (discrimination condition × TBS interaction (F_(6,122)_ = 1.032, *p* = 0.408, η²_p_ = 0.05) and across trials (block × TBS interaction: *F*_(13.835,281.303)_ = 0.600, *p* = 0.863, η²_p_ = 0.03). All groups performed well and reached about 87% correct responses in each of the three discrimination conditions at the end of the learning session. TBS groups did also not differ in the number of missed trials (*F*_(3,61)_ = 1.176, *p* = 0.326, η²_p_ = 0.06) or their reaction times (*F*_(3,61)_ = 1.424, *p* = 0.244, η²_p_ = 0.07), thus ruling out any differences in speed-accuracy trade-offs between groups.

### Devaluation phase

**Figure 3:**
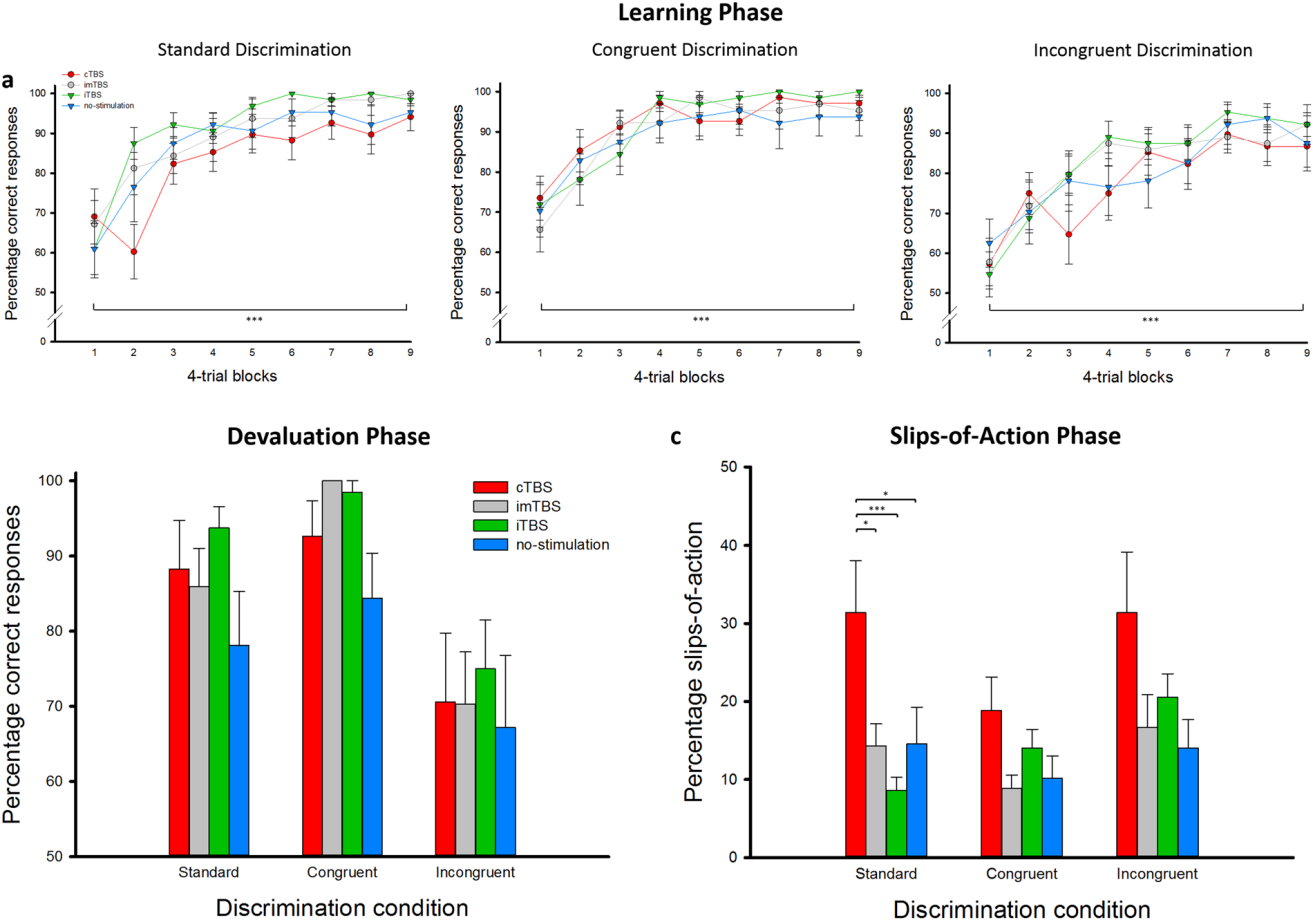
Performance in the Instrumental Learning Task (ILT). (**a**) Participants learned the associations between the fruit stimulus pairs and the according responses very well, independent of TBS condition and across discrimination conditions. (**b**) Performance in the devaluation phase was worse in the incongruent discrimination condition compared to the congruent and standard condition. TBS groups did not differ in their performance. (**c**) In the critical slips-of-action phase, cTBS led to a significant increase in the percentage of slips-of-actioncompared to the imTBS and iTBS groups as well as the no-stimulation group. Inhibition of the ilPFC favored the use of the habitual system, in particular in the standard discrimination condition that could be completed using either the habitual or the goal-directed system. Error bars indicate SEM. *p < 0.05. ***p < 0.001.

Participants performed also well in the devaluation phase, with each group reaching at least 76% correct responses (Fig. 3b). Again, performance depended on the discrimination condition (*F*_(1.521,92.765)_ = 20.629, *p* < 0.001, η²_p_ = 0.25), with better performance in the congruent condition compared to both the standard and the incongruent condition (post-hoc tests: *p* = 0.004 and *p* < 0.001, respectively) and in the standard compared to the incongruent condition (post-hoc test: *p* < 0.001). TBS condition did not affect task performance (*F*_(3,61)_ = 1.345, *p* = 0.270, η²_p_ = 0.06) and did not interact with the discrimination condition (*F*_(4.562,92.765)_ = 0.361, *p* = 0.859, η²_p_ = 0.02). As during learning, TBS had no effect on missed trials (*F*_(3,41)_ = 1.976, *p* = 0.127, η²_p_ = 0.09). There was, however, a main effect of TBS condition on reaction times (*F*_(3,60)_ = 3.048, *p* = 0.035, η²_p_ = 0.13), indicating slower responses in the no-stimulation group compared to the imTBS group (post-hoc test: *p* = 0.038; all other post-hoc comparisons: *p* > 0.125).

### Slips-of-action phase

The predominance of goal-directed action vs. habitual responding was revealed in the slips-of-action phase. Overall, participants’ performance differed between discrimination conditions (*F*_(2,122)_ = 8.090, *p* = 0.001, η²_p_ = 0.12), showing less slips of action in the congruent condition than in the standard condition (post-hoc test: *p* = 0.018), which can be under both habitual and goal-directed control^20,21^, or in the incongruent condition (post-hoc test: *p* = 0.001) which is assumed to rely mainly on the habit system^20^. Most importantly, however, there was a main effect of TBS condition on the slips of action (*F*_(3,61)_ = 3.547, *p* = 0.020, η²_p_ = 0.15). Participants in the cTBS condition showed a significantly higher percentage of slips compared to participants in the imTBS (post-hoc test: *p* = 0.009), the iTBS (post-hoc test: *p* = 0.017) or the no-stimulation condition (post-hoc test: *p* = 0.008), with all other groups being comparable in performance (all *p* > 0.780). As shown in Fig. 3c, cTBS doubled the percentage of slips compared to the other groups. Moreover, there was a TBS condition × discrimination condition interaction (*F*_(6,122)_ = 2.325, *p* = 0.037, η²_p_ = 0.12), suggesting that TBS primarily affected performance in the standard discrimination trials (*F*_(3,61)_ = 4.880, *p* = 0.004, η²_p_ = 0.19), with a significantly higher percentage of slips in those trials in the cTBS condition compared to the imTBS, iTBS and no-stimulation conditions (post-hoc tests: all *p* < 0.010; imTBS vs. iTBS: *p* = 0.376; imTBS vs. no-stimulation: *p* = 0.968; iTBS vs. no-stimulation: *p* = 0.355). In contrast, we found no effects of TBS in the congruent (*F*_(3,61)_ = 2.290, *p* = 0.087, η²_p_ = 0.10) or incongruent discrimination trials (*F*_(3,61)_ = 2.226, *p* = 0.090, η²_p_ = 0.10). Importantly, when participants made a slip, they did so by pressing the button that had previously been correct. Specifically, in each group the percentage of “correct” button presses during slips was at least 83%, which is comparable to percentage of correct responses for still valuable items (at least 85% in each group). Also, there was no effect of TBS on the percentage of formerly correct responses to devalued and still valuable fruits (*F*_(3,59)_ = 1.167, *p* = 0.330, η²_p_ = 0.06 and *F*_(3,61)_ = 1.869, *p* = 0.144, η²_p_ = 0.08, respectively).

In addition to the mere percentage of slips, we also calculated a difference score reflecting the relative use of goal-directed vs. habitual behavior^20,21,29^. The pattern of results for the differences score generally resembled the pattern observed for the percentage of slips (see supplementary results).

In order to test whether the larger amount of slips in the standard discrimination condition observed in the cTBS group was caused by less explicit knowledge of S-O-R associations, we performed an ANCOVA using performance in the ILT questionnaire as a covariate. While we found a significant effect of the ILT questionnaire on the slips of action (*F*_(1,60)_ = 15.259, *p* < 0.001, η²_p_ = 0.20), importantly the effect of TBS condition in the standard discrimination condition remained significant (*F*_(3,61)_ = 4.750, *p* = 0.005, η²_p_ = 0.19), indicating that cTBS led to more slips compared to imTBS (post-hoc test: *p* = 0.004), iTBS (post-hoc test: *p* = 0.001) and no-stimulation (post-hoc test: *p* = 0.012).

Again, TBS groups did not differ in missed trials (*F*_(3,61)_ = 0.404, *p* = 0.751, η²_p_ = 0.02) or in their reaction times (*F*_(3,61)_ = 0.776, *p* = 0.512, η²_p_ = 0.04).

### Contingency questionnaires

The different TBS conditions did neither affect participants’ ability to explicitly reproduce the contingencies between the fruit stimuli and the button responses (all *F*s < 1.103, all *p*s > 0.355) nor the certainty of their answers (all *F*s < 1.186, all *p*s > 0.323; see Table 1).

**Table 1:** Performance and certainty ratings in the contingency questionnaires.

	cTBS	imTBS	iTBS	No-stimulation
Percentage correct responses				
Standard discrimination condition	94.12 ± 3.48	93.75 ± 3.00	98.96 ± 1.04	96.88 ± 3.12
Congruent discrimination condition	98.04 ± 1.96	98.96 ± 1.04	100 ± 0.00	94.79 ± 3.64
Incongruent discrimination condition	95.10 ± 2.77	97.92 ± 2.08	95.83 ± 4.17	90.63 ± 4.80
Certainty ratings				
Standard discrimination condition	91.96 ± 3.99	94.06 ± 2.30	96.56 ± 2.12	87.29 ± 5.02
Congruent discrimination condition	99.02 ± 5.73	97.08 ± 1.17	98.85 ± 1.15	95.31 ± 2.74
Incongruent discrimination condition	93.14 ± 4.64	93.96 ± 2.40	96.88 ± 1.67	93.75 ± 3.79

Data represent mean ± SEM. cTBS, continuous theta burst stimulation; imTBS, intermediate theta burst stimulation; iTBS, intermittent theta burst stimulation.

### Control variables

Importantly, the TBS groups did not differ in mean stimulation intensity (*F*_(2,46)_ = 0.080, *p* = 0.992, η²_p_ < 0.01) or perceived discomfort after stimulation (*F*_(2,46)_ = 0.694, *p* = 0.505, η²_p_ = 0.03).

The four experimental groups did further not differ in most of the measures of personality traits and behavioral tendencies associated with decision-making processes which were measured at the beginning of the experiment, i.e. before the TBS manipulation (all *F*s < 1.665; all *p*s > 0.186; see Table 2).

**Table 2:** Scores in the control measures.

	cTBS	imTBS	iTBS	No-stimulation
TBS Intensity	45.76 ± 1.61	45.88 ± 2.13	45.56 ± 1.38	/
TBS Unpleasantness	6.79 ± 0.35	6.19 ± 0.47	6.13 ± 0.52	/
BDI	4.59 ± 1.55	3.44 ± 2.09	4.06 ± 1.67	5.44 ± 1.00
NEO FFI				
Neuroticism	28.41 ± 1.88	28.31 ± 2.30	27.44 ± 2.28	31.63 ± 2.00
Extraversion	42.47 ± 0.97	39.06 ± 2.03	42.56 ± 1.29	42.94 ± 1.65
Openness	43.71 ± 1.20	43.88 ± 1.04	43.38 ± 1.20	43.69 ± 0.97
Agreeableness	44.53 ± 1.74	43.63 ± 1.40	46.13 ± 1.51	44.06 ± 1.52
Conscientiousness	43.24 ± 1.43	43.81 ± 1.61	46.56 ± 1.58	46.63 ± 1.43
BIS/BAS scales				
BIS	15.35 ± 0.79	16.50 ± 1.35	16.69 ± 0.73	15.38 ± 1.14
BAS overall score	21.71 ± 0.86	23.44 ± 1.03	22.31 ± 0.60	21.63 ± 1.30
BAS drive	7.65 ± 0.36	7.31 ± 0.55	7.50 ± 0.35	6.88 ± 0.52
BAS fun seeking	6.41 ± 0.35	7.75 ± 0.48	7.06 ± 0.31	6.94 ± 0.41
BAS reward responsiveness	7.65 ± 0.49	8.63 ± 0.46	7.75 ± 0.48	7.81 ± 0.56
BIS 15				
Non-planning	10.94 ± 0.51	11.25 ± 0.81	10.50 ± 0.76	9.69 ± 0.81
Motor	11.65 ± 0.56	11.50 ± 0.82	11.13 ± 0.46	10.63 ± 0.46
Attentional	9.59 ± 0.68	9.81 ± 0.56	8.06 ± 0.47	9.50 ± 0.71
Overall score	32.18 ± 1.34	32.56 ± 1.68	29.69 ± 1.35	29.81 ± 1.37

Data represent mean ± SEM. cTBS, continuous theta burst stimulation; imTBS, intermediate theta burst stimulation; iTBS, intermittent theta burst stimulation; BDI, Beck Depression Inventory; NEO FFI, NEO Five Factor Inventory; BIS/BAS scales, Behavioral Inhibition/Behavioral Activation System scales; BIS 15, Barratt Impulsiveness Scale.

## Discussion

Extensive evidence from human and rodent studies demonstrates that behavior can be controlled by a deliberative, goal-directed system or a more reflexive, habitual system^2,4,30–33^. Over the past decades, a number of studies identified a network of brain areas relevant for the goal-directed vs. habitual control of behavior, including the orbitofrontal cortex, striatal and thalamic areas as well as the amygdala^8,11,12,34–38^. However, only very recently the ilPFC, a region which has previously been implicated in cognitive control processes^39–41^, has been linked to goal-directed and habitual behavioral control^13^. Using neuronavigated TBS, we show that the ilPFC indeed plays a causal role in the goal-directed control of behavior. Specifically, our findings show that cTBS over the ilPFC leads to a breakdown of the goal-directed control of action, paralleled by an increase in habitual responding. This effect could not be explained by differences in initial learning performance, speed-accuracy trade-offs, or personality traits relevant for decision-making.

The predominance of the goal-directed vs. the habitual system was reflected in the slips of action in an instrumental learning task. Previous studies using this task showed that goal-directed action relies on the ventromedial PFC and its connection with the dorsomedial striatum, whereas habitual responding, indicated by a higher number of slips, was linked to the connection between premotor areas and the dorsolateral striatum^19,20^; in line with rodent studies and neuroimaging studies using other tasks to investigate the neural basis of goal-directed and habit behavior^4,5,9,10,12,42^. However, the focal TBS applied here was not directed at one of those areas but specifically at the ilPFC. Our results are in line with recent data suggesting that the ilPFC acts as an arbitrator that allocates behavioral control to the goal-directed vs. habit system^13^. More specifically, the ilPFC has been postulated to track the reliability of the goal-directed and habit systems and to allocate control by modulating the degree to which the habit system can guide behavior^13^. We chose the stimulation site exactly according to the recent study showing such a role of the ilPFC. Thus, although the task used here does not allow a direct assessment of the arbitration process, the observed increase in slips-of-action after cTBS may be due to a modulation of this arbitrator region. Whereas the previous evidence linking the ilPFC to the balance of goal-directed action and habitual responding was based on correlational data^13^, our data provide first evidence for a causal role of the ilPFC in the modulation of goal-directed and habitual behavior. Because the ilPFC was suggested to allocate behavioral control mainly via the modulation of the habit system^13^, we further suggest that cTBS may have interfered mainly with the capacity of the ilPFC to downregulate the habit system when behavior should be under goal-directed control.

Assuming that the arbitration mechanism works mainly through the modulation of the habit system when the ilPFC deems behavior should be driven by the goal-directed system, this could explain why we did not find an effect of iTBS. In contrast to cTBS, iTBS should have led to increased activity of the ilPFC^18^. Yet if intact ilPFC functioning is already sufficient to control the habit system and if the ilPFC does not strengthen goal-directed control, one would not necessarily expect a further increase in goal-directed action after iTBS of the ilPFC. In line with this idea, there were only relatively few slips-of-action after sham imTBS and in the no-stimulation control group, suggesting that there was not much room for further improvement of goal-directed control by iTBS. However, in addition to this intriguing idea, we cannot fully exclude the alternative possibility that our data might also be explained by a more direct involvement of the ilPFC in goal-directed behavior. The ilPFC is directly connected to areas involved in goal-directed action^43,44^. Moreover, the role of the lateral PFC in inhibition and executive control, i.e. in cognitive functions that are essential for goal-directed behavior, is well established^14,39–41^. It is, however, important to underline that our findings cannot be interpreted as a simple modulation of inhibitory control. If this were the case, we should see slips-of-action in all discrimination trials and in particular in those for which acquisition performance was best (and responses therefore most difficult to suppress). However, slips of action were mainly observed in incongruent trials, thought to rely mainly on the habit system^20^, although acquisition performance was worse in those trials than in congruent or standard trials. Moreover, cTBS did not affect performance in congruent or incongruent trials but specifically in standard discrimination trials, in which goal-directed and habitual systems compete for control^19^. This specificity of our effects argues against a modulation of general inhibitory control but instead suggests a specific alteration of the goal-directed control of behavior.

Since we applied TBS before the ILT, it could have affected both the initial acquisition of the associations and the goal-directed vs. habitual control in the slips-of-action test. Because standard and congruent trials can be solved by both systems equally well and performance in the incongruent trials is thought to rely on the habit system anyway^19,20,29^, performance should be comparable no matter which system guides behavior. Nevertheless, a differential recruitment of the goal-directed and habit systems during acquisition may translate into more slips in the slips-of-action test and we cannot conclude that cTBS affected exclusively the performance in the slips-of-action test. Future studies using fMRI in combination with TBS may be helpful to assess the contribution of goal-directed and habit systems during task acquisition and tests of behavioral flexibility. It is, however, important to note that there were no differences in learning or devaluation performance between groups. Moreover, while the random assignment of participants to groups should have made baseline differences in the tendency towards goal-directed and habitual responding differences rather unlikely and groups did not differ in their performance in the short training session, it should be noted that, as in every between-subjects design, such baseline differences cannot be ruled completely ruled out.

It is important to note that studies report considerable individual differences in response sensitivity to TBS protocols, indicating that results of TBS studies need to be interpreted with caution^45–48^. These differences probably occur due to a multitude of intra- and interindividual factors including circadian influences, cortical network activity, genetic variability, age or developmental factors^45^. Evidence for this response variability primarily comes from research in motor areas, as MEPs allow for a direct assessment of stimulation effects. As studies on prefrontal cortical function lack a comparable read-out, the amount of variability in participants’ responses remain unclear. However, several studies using TBS on prefrontal areas report reliable effects on behavior^49–52^. A recent review and meta-analysis^27^ concludes that TBS over prefrontal areas exerts a small but consistent effect on behavior. Given these findings and the medium to large effects of our main results, we are confident that our data add to the accumulating evidence of the efficacy of prefrontal TBS.

In sum, we show that cTBS directed specifically at a region of the ilPFC, previously implicated in cognitive control processes and in balancing the allocation of behavioral control between the goal-directed and the habit system^13^, renders behavioral responding less goal-directed. Although the ilPFC is most likely not acting in isolation, but communicating extensively with other regions supporting goal-directed or habitual behavior, our findings demonstrate a causal role of the ilPFC in the goal-directed control of behavior. These findings shed light on the neural mechanisms underlying the balance of deliberate, goal-directed action and efficient but rather rigid habitual responding. Moreover, our findings provide novel insights into how goal-directed action may break down in disorders such as addiction or OCD that are characterized by impaired goal-directed control^29,53,54^.

## Materials and Methods

### Participants and Experimental Design

Sixty-five healthy men and women between 19 and 32 years of age participated in this experiment (mean age ± SEM: 24.52 ± 0.36 years, 35 women). Exclusion criteria were checked in a standardized phone interview prior to testing and included current physical or mental conditions, medication or drug intake, a life-time history of any neurological disorder and pregnancy in women as well as any contraindications for MRI and TMS, including a history of epilepsy. All participants gave written informed consent prior to testing and received a moderate monetary compensation. The study protocol was in line with the Declaration of Helsinki and approved by the ethics committee of the Faculty of Psychology and Human Movement Sciences of the University of Hamburg (26 2015 Bogdanov).

We used a double-blind, sham controlled, between-subjects design, in which participants were randomly assigned to one of four experimental conditions: continuous, intermediate, or intermittent theta burst stimulation (TBS) of the right ilPFC or a no-stimulation control condition (cTBS: 8 men, 9 women; imTBS: 7 men, 9 women; iTBS: 7 men, 9 women; no-stimulation: 8 men, 8 women). The inclusion of these four conditions allowed us to test potential stimulating and inhibiting effects and to rule out any unspecific effects of TBS.

We would like to note that we had initially tested 48 participants. Due to reviewer concerns about this sample size, we tested 17 additional participants. The data presented here refer to the complete sample of 65 participants. Importantly, however, all relevant main effects had already been significant in the original sample of 48 participants.

### MRI acquisition

For each participant, a high-resolution T1-weighted anatomical MRI image was acquired using a 3 T Skyra scanner (Siemens) equipped with a 32-channel head coil with the following parameters: TR = 2.5 s, TE = 2.12 ms, 256 slices, voxel size = 0.8 × 0.8 × 0.9 mm.

### Neuronavigated TMS application

Using the anatomical MRI image, the individual position of each participant’s right ilPFC for the application of TMS was determined using a stereotaxic frameless Brainsight neuronavigation system (Rogue-Reseach, Canada). First, a 3D-reconstruction of each participant’s brain was built. We then specified the right ilPFC as the stimulation target. The exact stimulation site (MNI coordinates: x = 48, y = 35, z = −2) was chosen based on fMRI evidence pointing to the ilPFC as a critical structure in balancing goal-directed and habitual control of behavior^13^ (Fig. 1a). Finally, the coil position was adjusted according to the target and marked for later stimulation.

In order to obtain optimal stimulation intensity, we determined participants’ individual resting motor threshold (RMT). Therefore, the coil was moved over the hand area of the left motor cortex. Motor potentials were recorded from the first dorsal interosseus muscle of the right hand using disposable Ag/AgCL surface electrodes and a micro 1401-mkII data acquisition unit with a 1902 pre amplifier by Cambridge Electronic Design (UK). RMT was defined as the stimulator intensity necessary to evoke muscle responses greater than 50 µV in at least 5 of 10 consecutive pulses. Depending on the individual stimulation intensity, TBS was delivered at 80% RMT using either a Magstim Super Rapid² or a Magstim Super Rapid² Plus stimulator (The Magstim Company Ltd, UK) and a 70 mm figure-of-eight coil. Following the standard protocols for TBS established for the motor cortex^18^, participants received a series of bursts of 3 magnetic pulses (pulse triplets) at a frequency of 50 Hz which were repeated at a rate of 5 Hz (i.e., 5 pulse triplets per second). In total, each participant received 600 magnetic pulses. Depending on the experimental condition, these pulse triplets were delivered using one of three different stimulation protocols (Fig. 1b). In the continuous stimulation protocol (cTBS), pulses were delivered continuously for a duration of 40 s. In the intermittent stimulation protocol (iTBS), pulses were delivered in intervals of 2s-stimulation-trains followed by a 8s-pause for a total of 190 s. In the intermediate stimulation protocol (imTBS), pulses were delivered in intervals of 5s-stimulation-trains followed by a 10s-pause for a total of 110 s. It has been shown, that cTBS leads to a deactivation of the targeted brain region for up to 60 minutes, whereas iTBS leads to an activation. ImTBS, however, has no such effect and represents thus a sham stimulation^18,28^. The coil was positioned tangential to the previously marked location on the head and manually held throughout the stimulation. Noticeable side effects consisted of muscle twitching in the face and neck, which participants reported to be moderately unpleasant.

### Instrumental Learning Task

About 15 minutes after TBS, participants completed a modified instrumental learning task (ILT)^20,21,29,55^ that allowed us to assess the goal-directed vs. habitual control of behavior. This task consisted of three phases: the learning phase, the devaluation phase, and the slips-of-action phase. During these phases, participants were presented with six different stimulus pairs consisting of fruit pictures (Fig. 2). These fruit pictures could serve either as discriminative cue stimuli (when depicted on top of a closed box) or as outcomes (when depicted within an open box). The stimulus pairs were linked by responses (button presses) that, if correct, would lead from a cue to an outcome. If participants acted in a goal-directed manner, they would be able to form stimulus-outcome-response (S-O-R) associations whereas habitual behavior would be reflected in the formation of simpler stimulus-response (S-R) associations. There was a total of six stimulus pairs with two pairs belonging to one of three different discrimination conditions (Fig. 2a): congruent, incongruent, and standard. In the *congruent* condition, cue and outcome were the same fruit. Participants would thus only need to associate one fruit stimulus with the according response. In the *incongruent* condition, cue and outcome were different fruits. However, these stimulus pairs reversed their role across trials, demanding opposite responses depending on which fruit served as the cue (e.g., if a banana cue led to an orange by pressing the left arrow-key then an orange cue would lead to a banana by pressing the right arrow-key). It is argued that in this condition, goal-directed behavior leads to a response conflict because the two fruit stimuli are associated with opposite responses. For example, a banana should activate the “banana-orange-left” (S-O-R) association. In this case, however, the banana would additionally activate the “banana-right” (O-R) association learnt in trials in which the orange served as a cue. To avoid this response conflict, participants should rely on simple S-R (“banana-left”, “orange-right”) associations. Thus, the incongruent discrimination condition is usually used as a baseline measure of habitual behavior^19,29,55^. Finally, in the *standard* discrimination condition, a correct response towards the cue would lead to a distinct outcome fruit that was not otherwise used in the experiment. Participants could use either the habitual or the goal-directed system to form associations in these trials^20,56^. However, flexible adaption of behavior in the later slips-of-action phase critically depends on functional S-O-R associations (i.e. goal-directed behavior).

In the initial *learning phase* (Fig. 2b), participants were asked to learn associations between the discriminative cue stimulus, the appropriate response and the outcome for each stimulus pair by trial and error. In each trial, they were presented with a picture of a fruit on top of a closed box and were asked to open the box by pressing either the left or the right arrow-key on a keyboard. If the response was correct, the box opened to reveal the fruit outcome. If incorrect, the open box was empty. Correct responses also earned points depending on reaction times (5 points for reaction times within 0–1 second; 4 points for 1–2 seconds; 3 points for 2–3 seconds; 2 points for 3–4 seconds; 1 point for 4–5 seconds). Participants were explicitly instructed to respond as fast as possible and had 5 seconds to respond before the trial was terminated and the next trial started. They were informed that a higher total score would convert into a higher monetary reward in order to motivate proper task performance. Importantly, participants could use both systems to complete the learning phase, forming either simple S-R (i.e. habitual) or more complex S-O-R (i.e. goal-directed) associations. In total, participants completed 9 learning blocks with each block consisting of 12 trials (4 trials per discrimination condition).

In the subsequent *outcome devaluation phase* (Fig. 2c), participants saw two open boxes containing fruits that were previously shown as outcomes but were associated with opposite responses (i.e., one of the depicted fruits followed a right button press, the other fruit followed a left button press). However, one of these formerly rewarded fruits was now devalued, as indicated by a red cross covering it. Participants were informed that the devalued fruit would no longer earn any points and that they should press the arrow-key that was associated with the other, still valuable fruit, only. There was a total of 12 trials, each former outcome was devalued twice. No feedback was shown to the participants in this phase. This outcome devaluation phase was included primarily to get the participants used to responding to former rewards instead of discriminative cue stimuli and to familiarize participants with the devaluation.

The critical *slips-of-action phase* (Fig. 2d) consisted of 6 blocks of 24 trials each. At the beginning of each block, participants were presented with an overview depicting 6 open boxes showing all fruits that served as rewarded outcomes in the learning phase. Importantly, two of these fruits were now marked with a red cross indicating that they were now devalued. This overview was shown for 10 seconds. Similar to the learning phase, participants were then presented with single closed boxes with fruits on them. They were instructed to earn points by pressing the correct button associated with the fruit cue on the box only when the outcome fruit that would appear in the box was still valuable. If, however, the response would lead to a devalued outcome, participants should refrain from pressing any button at all. Any response towards a cue leading to a now devalued fruit would result in a subtraction of 2 points from the total score. Again, no feedback was provided to the participants. If participants responded in a goal-directed manner (i.e. relying on S-O-R associations), responses in each trial should be adapted to the value of the associated outcome fruit. If, however, participants responded more habitually (i.e. relying on S-R associations), they should show slips of action (i.e., responses to stimuli even when the outcome fruit is devalued). When the participants had completed the ILT, they were asked to fill in three questionnaires that tested their explicit knowledge about the stimulus-response, response-outcome and stimulus-outcome contingencies for each fruit pairing. In addition, participants also had to indicate their response certainty.

### Procedure

The individual MRI scan was acquired several days or weeks before behavioral testing. Upon their arrival at the lab on the testing day, participants signed the informed consent form before completing German versions of the Beck Depression Inventory^57^, the Behavioral Inhibition/Behavioral Activation System scale^58^, the Barratt Impulsiveness Scale^59^ and the NEO Five-Factor Inventory^60^. These questionnaires allowed us to control for personality traits and behavioral tendencies that are relevant in decision-making processes. For participants in the TBS groups, individual stimulation site and resting motor threshold were determined. Participants then received the ILT instructions and completed a training sequence in which they were presented with all three phases of the ILT using different fruits than in the main experiment. This was done to familiarize participants with the task and to avoid possible stimulation-induced differences in task comprehension. After training, the experimenter left the room while stimulation was applied over the right ilPFC by a different experimenter, ensuring double-blindness. Participants then completed the ILT, including the contingency questionnaires. For the no-stimulation group, the ILT began shortly after training. In general, participants completed the task within 30 minutes. At the end of the experiment, participants were asked to rate the unpleasantness of the stimulation procedure on a scale from 1 to 10. Finally, all participants were debriefed and received a monetary compensation.

### Data Analysis

For the learning and the devaluation phase, we calculated the percentage of correct responses as the primary measure of task performance. Reaction times were analyzed for the learning phase only. For the slips-of-action-phase, we focused on the percentage of slips. In addition, we calculated a difference score that reflects goal-directed behavior more generally by taking into account, that goal-directed behavior is also reflected in correct responses to valuable outcomes. In line with previous studies^20^, this score is calculated by subtracting incorrect responses (i.e., pressing a button when the outcome was devalued) from correct responses (i.e., correct button presses for valuable outcomes and no button presses for devalued outcomes). We also corrected for missing button presses for still valuable outcomes. This score ranges from −100 to 100 with higher numbers indicating more goal directed behavior and lower numbers more habitual responding^20^. Data were analyzed using a mixed-design ANOVA with number of blocks (9 for analyses of the learning phase only) and discrimination condition (standard vs. congruent vs. incongruent) as within-subject factors and TBS condition (continuous vs. intermediate vs. intermittent vs. no-stimulation) as between-subjects factor. If necessary, Greenhouse-Geisser correction was applied. Significant main or interaction effects were further pursued by appropriate *post-hoc* tests that were corrected for multiple comparisons, if required. All reported *p* values are two-tailed. Effect sizes are reported as partial η².

### Data availability

The datasets generated during and/or analyzed during the current study are available from the corresponding author on reasonable request.

## Electronic supplementary material

Supplemental material
