# Supplementary material for: Causal role of the inferolateral prefrontal cortex in balancing goal-directed and habitual control of behavior
Source: Sci Rep. 2018 Jun 20;8:9382. doi: 10.1038/s41598-018-27678-6 (PMC6010441; doi:10.1038/s41598-018-27678-6)
Supplement: Supplementary file 1 — Supplemental material [file 41598_2018_27678_MOESM1_ESM.docx]

***Supplemental information***

**Causal role of the inferolateral prefrontal cortex in balancing goal-directed and habitual control of behavior**

Mario Bogdanov^1^, Jan E. Timmermann^2^, Jan Gläscher^3^, Friedhelm C. Hummel^2,4^ and Lars Schwabe^1^

^1^ Department of Cognitive Psychology, Institute for Psychology, University of Hamburg, Germany;

^2^ Department of Neurology, University Medical Center Hamburg-Eppendorf, 20246 Hamburg

^3^ Institute for Systems Neuroscience, University Medical Center Hamburg-Eppendorf, 20246 Hamburg

^4^ Defitech Chair of Clinical Neuroengineering, Swiss Federal Institute of Technology (EPFL), 1202 Geneva, Switzerland

**Supplementary Results**

Difference score

In addition to the mere percentage of slips (i.e. responses towards devalued outcomes), we also calculated a difference score, reflecting the relative use of goal-directed vs. habitual behavior by subtracting more habitual responses (i.e. slips and incorrect non-responses towards valuable outcome) from goal-directed responses (i.e. correct responses towards valuable outcomes and correct non-responses towards devalued outcomes[^1-3^](#_ENREF_1). The general pattern of results for the difference score resembled the results for the percentage of slips. Performance was affected by the discrimination condition (*F*_(2, 122)_ = 20.018, *p* < .001, η²_p_ = .25), as participants showed more goal-directed behavior in the standard discrimination condition compared to the incongruent condition (post-hoc test: *p* < .001) as well as in the congruent discrimination condition compared to both the standard and the incongruent condition (post-hoc tests: *p* = .026 and *p* < .001, respectively; see Figure S1). There was a main effect of TBS condition (*F*_(3, 61)_ = 2.749, *p* = .050, η²_p_ = .12), indicating less goal-directed behavior in the cTBS group compared to both the imTBS and the iTBS group (post-hoc tests: *p* = .022 and *p* = .014, respectively) and a trend compared to the no-stimulation group (*p* = .053), while there was no difference between the other conditions (imTBS vs. iTBS: *p* = .865; imTBS vs. no-stimulation: *p* = .713; iTBS vs. no-stimulation: *p* = .591). There was no discrimination condition × TBS condition interaction (*F*_(6, 122)_ = 1.533, *p* = .173, η²_p_ = .07). However, looking at each discrimination condition, TBS again seemed to affect primarily the standard discrimination trials (*F*_(3, 61)_ = 3.673, *p* = .017, η²_p_ = .15), leading to less goal-directed behavior in the cTBS group compared to the imTBS group (*p* = .041), the iTBS group (*p* = .002) and the no-stimulation group (*p* = .039). There were no differences between the other groups (imTBS vs. iTBS: *p* = .261; imTBS vs. no-stimulation: *p* = .978; iTBS vs. no-stimulation: *p* = .272). In contrast, there were no effects of TBS condition in the congruent or incongruent trials (both *F* < 2.451, both *p* > .072).

**Table S1. Percentage of missed trials per experimental phase**

|  |  | **cTBS** | **imTBS** | **iTBS** | **No-stimulation** |
| --- | --- | --- | --- | --- | --- |
| Learning phase | |  |  |  |  |
|  | Standard discrimination | 0.00 ± 0.00 | 0.00 ± 0.00 | 0.00 ± 0.00 | 1.56 ± 1.56 |
|  | Congruent discrimination | 0.00 ± 0.00 | 0.00 ± 0.00 | 0.00 ± 0.00 | 1.74 ± 1.74 |
|  | Incongruent discrimination | 0.00 ± 0.00 | 0.00 ± 0.00 | 0.00 ± 0.00 | 2.08 ± 1.73 |
| Devaluation phase | |  |  |  |  |
|  | Standard discrimination | 4.41 ± 3.21 | 0.00 ± 0.00 | 1.56 ± 1.56 | 10.94 ± 6.44 |
|  | Congruent discrimination | 2.94 ± 2.01 | 0.00 ± 0.00 | 0.00 ± 0.00 | 10.94 ± 5.58 |
|  | Incongruent discrimination | 4.41 ± 4.41 | 0.00 ± 0.00 | 3.13 ± 2.13 | 9.38 ± 6.80 |
| Slips-of-action phase | |  |  |  |  |
|  | Standard discrimination | 4.00 ± 1.18 | 4.17 ± 2.35 | 1.48 ± 0.45 | 3.21 ± 1.17 |
|  | Congruent discrimination | 1.96 ± 0.73 | 1.65 ± 0.95 | 0.61 ± 0.31 | 1.48 ± 0.74 |
|  | Incongruent discrimination | 6.78 ± 1.82 | 4.34 ± 1.90 | 6.08 ± 1.17 | 6.68 ± 1.71 |

Data represent mean ± SEM. cTBS, continuous theta burst stimulation; imTBS, intermediate theta burst stimulation; iTBS, intermittent theta burst stimulation.

**Table S2. Reaction times (ms) per experimental phase**

|  |  | **cTBS** | **imTBS** | **iTBS** | **No-stimulation** |
| --- | --- | --- | --- | --- | --- |
| Learning phase | |  |  |  |  |
|  | Standard discrimination | 853.37 ± 50.96 | 723.29 ± 27.13 | 753.97 ± 36.16 | 792.33 ± 35.99 |
|  | Congruent discrimination | 732.34 ± 33.05 | 682.32 ± 28.67 | 707.06 ± 33.52 | 754.74 ± 41.69 |
|  | Incongruent discrimination | 922.32 ± 58.68 | 805.78 ± 44.18 | 873.79 ± 50.39 | 945.33 ± 62.28 |
| Devaluation phase | |  |  |  |  |
|  | Standard discrimination | 1376.55 ± 152.97 | 1123.01 ± 86.81 | 1195.46 ± 107.56 | 1785.58 ± 216.55 |
|  | Congruent discrimination | 976.51 ± 61.56 | 942.44 ± 38.23 | 1090.30 ± 80.59 | 1358.87 ± 173.08 |
|  | Incongruent discrimination | 1583.82 ± 212.60 | 1349.77 ± 100.07 | 1368.46 ± 90.63 | 1652.04 ± 212.60 |
| Slips-of-action phase | |  |  |  |  |
|  | Standard discrimination | 761.72 ± 35.57 | 751.07 ± 45.65 | 729.70 ± 27.96 | 792.36 ± 33.19 |
|  | Congruent discrimination | 690.69 ± 30.33 | 702.44 ± 27.82 | 704.33 ± 23.45 | 777.52 ± 28.45 |
|  | Incongruent discrimination | 839.07 ± 38.80 | 826.23 ± 38.60 | 864.54 ± 41.24 | 887.97 ± 42.41 |

Data represent mean ± SEM. cTBS, continuous theta burst stimulation; imTBS, intermediate theta burst stimulation; iTBS, intermittent theta burst stimulation.

**References**

1 de Wit, S. *et al.* Corticostriatal connectivity underlies individual differences in the balance between habitual and goal-directed action control. *The Journal of Neuroscience* **32**, 12066-12075 (2012).

2 Gillan, C. M. *et al.* Disruption in the balance between goal-directed behavior and habit learning in obsessive-compulsive disorder. *American Journal of Psychiatry* **168**, 718-726 (2011).

3 de Wit, S. *et al.* Reliance on habits at the expense of goal-directed control following dopamine precursor depletion. *Psychopharmacology* **219**, 621-631 (2012).


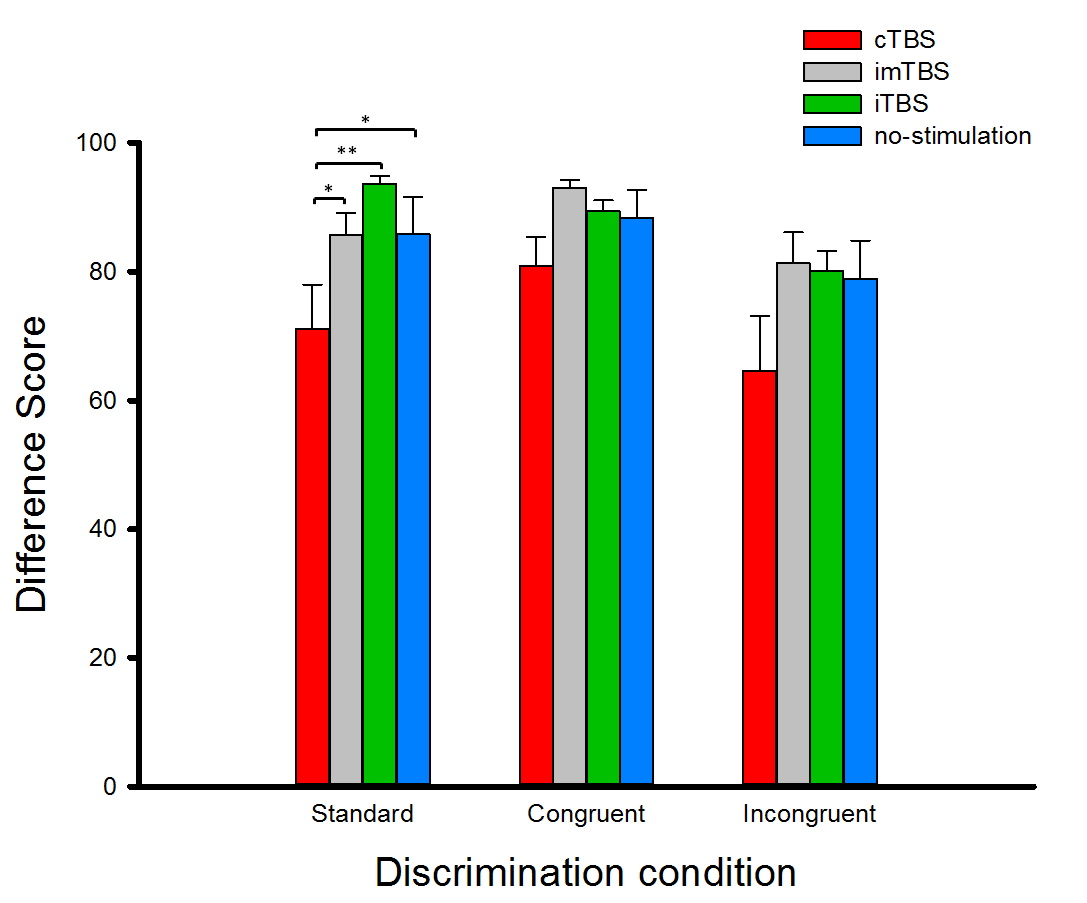


**Figure S1: Difference score.** Participants’ behavior in the cTBS condition was less goal-directed and more habitual compared to imTBS, iTBS and the no-stimulation group. This seemed to be the case especially in the standard discrimination trials, that could be completed using either the habitual or the goal-directed system. Error bars indicate SEM. *p < 0.05. **p < .01
